# A Case of Reversible Atrioventricular Block Potentially Associated with Atenolol-Induced Hyperkalemia

**DOI:** 10.7759/cureus.13151

**Published:** 2021-02-05

**Authors:** Kay Thi Oo, Hnin Wut Yee

**Affiliations:** 1 Internal Medicine, Interfaith Medical Center, Brooklyn, USA

**Keywords:** hyperkalemia, beta-blocker, end stage renal disease (esrd), second degree heart block, reversible heart block

## Abstract

Potassium is an extracellular ion that plays an important role in the electrophysiological function of the heart. Any change in the extracellular concentration of potassium can have a marked impression upon cardiac electrophysiology. Underlying kidney disease, certain medical conditions, dietary indiscretions, and medications can precipitate hyperkalemia. Drug-induced hyperkalemia is one of the most important causes of increased serum potassium in everyday clinical practice. Hyperkalemia can lead to various life-threatening dysrhythmias and if left untreated, it will ultimately cause ventricular arrhythmias and asystole. This case report describes an end-stage renal disease (ESRD) patient taking atenolol who presented with hyperkalemia and type II second degree atrioventricular (AV) block. He presented with hyperkalemia when atenolol was introduced and normalized when atenolol was discontinued. The heart block completely resolved after treatment of hyperkalemia.

## Introduction

Hyperkalemia is a potentially life-threatening condition that is defined as a serum potassium level above a reference range, usually greater than 5.0 mEq/L; severe hyperkalemia is often defined as a level greater than 6.0 mEq/L. Prescribed medications, over-the-counter drugs, and nutritional supplements that can affect the potassium levels are used by many patients. Although most of these products are well tolerated, drug-induced hyperkalemia may develop in patients with underlying renal impairment or other abnormalities in potassium handling [1]. In hospitalized patients, the incidence of hyperkalemia ranges from 1.3% to 10%, with a mortality rate of 1 per 1,000 patients [2]. Drug-induced hyperkalemia is one of the most important causes in everyday clinical practice and has been identified as a primary contributing factor of hyperkalemia in 35%-75% of hospitalized patients [2]. Beta-blockers are postulated to induce hyperkalemia by suppressing catecholamine stimulated renin release, decrease aldosterone levels and impair cellular uptake of potassium [3]. Beta-blocker can increase serum potassium level by 1 mmol/L or more in end-stage renal disease (ESRD) [2]. Hyperkalemia is more commonly seen with nonselective rather than with cardio-selective beta-blockers [2]. However, there is one reported case of atenolol-induced hyperkalemia in the medical literature [4]. The duration of hyperkalemia can be fairly prolonged with advanced chronic kidney disease, particularly when renally cleared beta-blockers such as atenolol, are administered [2].

There is one reported case of complete heart block induced by hyperkalemia in an ESRD patient that completely resolved to normal with hyperkalemia treatment but months later developed permanent right bundle branch block (RBBB) and left anterior fascicular block [5]. The uniqueness of our case is that although there can be various ranges of heart block induced by hyperkalemia, there is no reported case so far presenting with reversible atrioventricular (AV) block potentially associated with atenolol-induced hyperkalemia in a patient without documented heart disease.

## Case presentation

A 64-year-old Asian male with a past medical history of hypertension, type 2 diabetes mellitus, anemia, and ESRD on hemodialysis was referred by the dialysis center for evaluation after the patient was found to have a heart rate in the 30s and was brought to the emergency department (ED) by ambulance. He receives hemodialysis at an outpatient dialysis center three days a week, reported to be compliant, and did not miss any session. However, when he went to the dialysis center, he was found to have low blood pressure (BP) of 88/47 mmHg and a heart rate (HR) of 45 bpm. Leg elevation and inhalational oxygen via nasal cannula were provided but vitals rechecked after 30 minutes revealed BP 145/68 mmHg and HR 36/min. Therefore, emergency medical services (EMS) was activated, and the patient was brought to the ED. 

**Figure 1:**
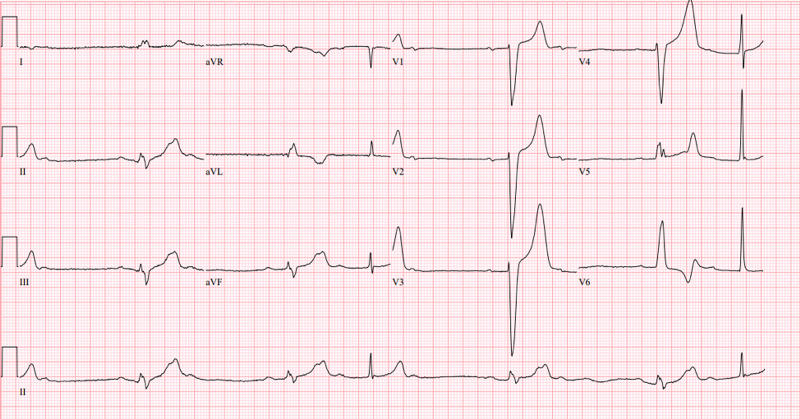
EKG changes before treatment

Vitals on triage in the ED were notable for BP 108/54 mmHg, HR 33 bpm, oxygen saturation 100% on 2 liters nasal cannula (NC). He was alert, awake, and answered questions appropriately. He was not aware of his slow heart rate, but he complained of feeling dizzy on and off over the past two days. He denied chest pain, palpitation, shortness of breath, and spells of blackout. Stat EKG was done which showed ventricular rate of 37 bpm, PR interval 284 ms, QRS duration of 154 ms, QTc 466 ms. Fingerstick blood glucose was 195 mg/dl. Further interpretation of EKG revealed second degree 2:1 AV conduction with wide QRS suggestive of type II second degree AV heart block and peaked T wake (Figure 1). This is not his first-time presentation with a similar picture. His past medical records showed that he was admitted three times over the past two years with a similar presentation of hyperkalemia and heart block, one time with first degree AV block, another two times with type I second degree AV block, all resolved to normal sinus rhythm after potassium was normalized. His medications list was significant for atenolol which was discontinued in his previous admissions but was again prescribed a few days before his presentation to the ED. 

His physical examination was unremarkable except for severe bradycardia and cool and clammy extremities. On the cardiac monitor, his heart rate was ranging between 30 bpm to 35 bpm. In view of his previous presentations and EKG findings, he was given calcium gluconate 1 gram slow intravenous (IV) stat, 10 units of IV regular insulin, 25 grams of dextrose 50%, and 12 milliliters of albuterol nebulizer while waiting for blood tests results. 

While receiving treatment, basic labs including plasma potassium were sent. The results came back with serum potassium of 8.7 mmol/L, plasma potassium of 8.6 mmol/L. Other chemistries were sodium 135 mmol/L, chloride 96 mmol/L, bicarbonate 23 mmol/L, blood urea nitrogen (BUN) 78 mg/dl, creatinine 14.54 mg/dl, glucose 192 mg/dl, magnesium 2.6 mg/dl, and phosphorous 7.3 mg/dl.

His heart rate showed slight improvement after receiving treatment. Nephrology team recommended emergency hemodialysis. He was also given 30 grams of Kayexylate orally, an additional 2 grams of calcium gluconate slow IV while awaiting hemodialysis set up. He received a total of four hours of hemodialysis with 1K bath for the first 2.5 hours followed by 2K bath for the remaining treatment. Two hours post-dialysis, potassium came back 3.3 mmol/L. 

**Figure 2:**
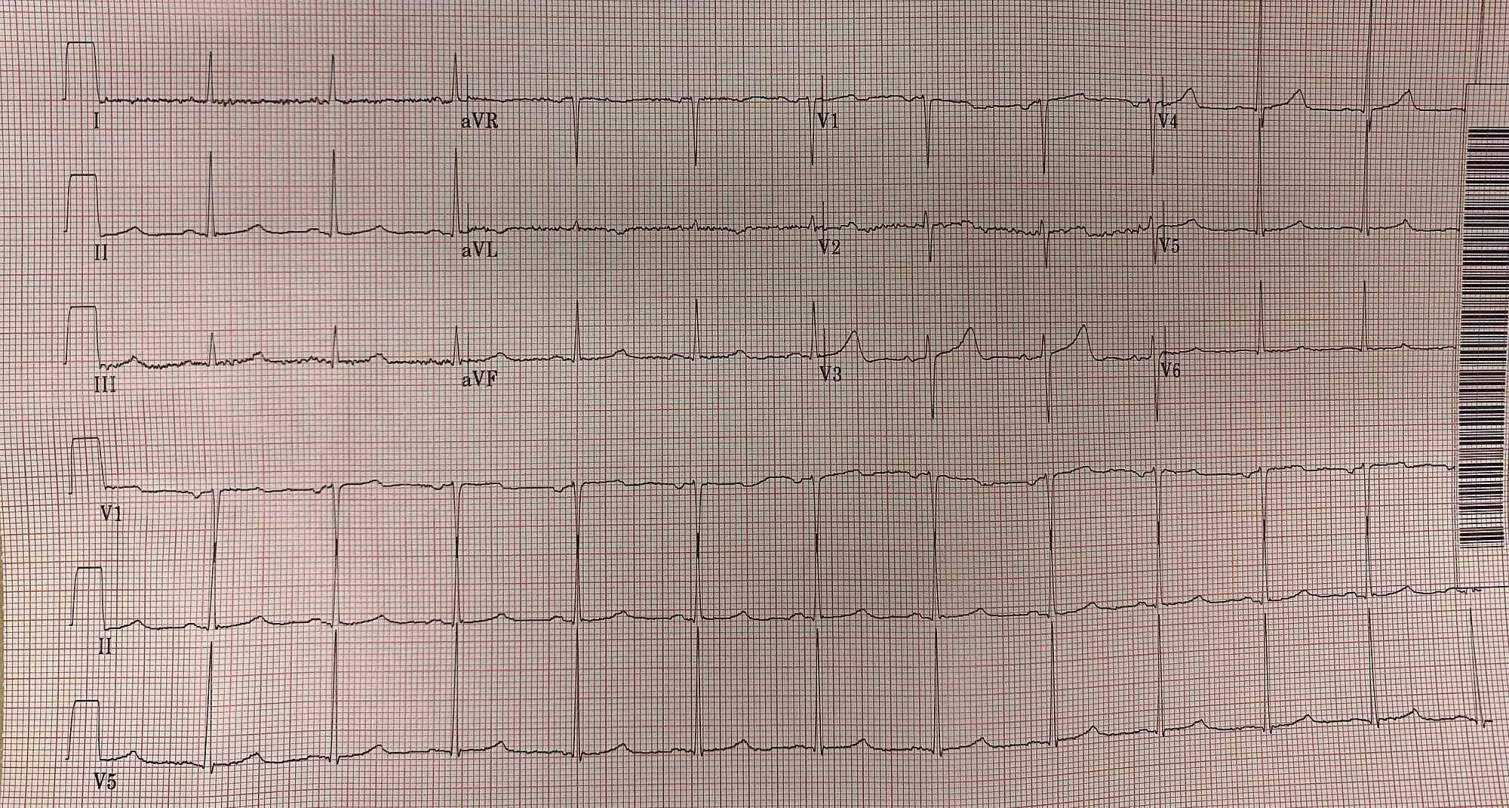
Normal sinus rhythm after treatment

The patient was admitted to the telemetry unit with nephrology on board. His hyperkalemia was thought to be secondary to atenolol considering his multiple presentations provoked by the reintroduction of atenolol. His cardiac enzyme was not elevated. Continuous cardiac monitoring was uneventful. At the time of discharge, his potassium level was 4.6 mmol/L. Beta-blocker was held throughout his hospital stay and also not continued upon discharge. His EKG on the day of his discharge demonstrated complete resolution of the previously seen type II second degree AV block pattern. EKG showed a rate of 72 bpm, PR interval 156 ms, normal sinus rhythm, and slightly prolonged QTc 462 ms (Figure 2).

## Discussion

This case depicts an unusual presentation of atenolol-induced hyperkalemia causing type II second degree heart block that resolves completely to normal after correction of hyperkalemia, and discontinuation of atenolol. In this patient, who is reported to have started taking atenolol a few days before presentation, it is reasonable to believe that his hyperkalemia could be induced by atenolol, and heart block could be contributed by atenolol-induced hyperkalemia. For some reason, even though atenolol was discontinued upon previous discharges because of similar presentation, it was again prescribed by different doctors. Contributing factors could be lost to follow up upon discharge, no dedicated primary doctor, language barrier to some extent even though language line was properly used, and lack of health knowledge. In view of his recurrent presentation of hyperkalemia-induced heart block, electrophysiologic studies would be warranted to rule out any underlying pathology. However, the disappearance of hyperkalemia-induced heart block with hyperkalemia treatment and discontinuation of atenolol, the absence of any other acute causes of AV block make it seem unlikely. The resolution of type II second degree heart block to sinus rhythm after treatment of hyperkalemia and hemodialysis also suggest that atenolol-induced hyperkalemia is responsible for this high-grade AV block. 

Hyperkalemia can lower cell-resting action potential and causes abnormal heart muscle function resulting in EKG findings initially with tall and tented T wave, loss of P wave, prolong PR interval, widened QRS complex, and eventually sine wave before the heart stops [6]. Varying degrees of heart block can also happen with this electrolyte disturbance but advanced heart blocks such as second- and third-degree AV blocks are generally found only in patients with underlying heart disease, heart failures, or preexisting conduction abnormalities. It was reasoned that underlying coronary artery or diffuse cardiac disease have already destroyed the AV node and His-Purkinje system to a certain extent that hyperkalemia can aggravate pre-existing diagnosed or undiagnosed conduction defects [7].

Beta-blocker can increase serum potassium level by 1 mmol/L or more in ESRD [2]. Hyperkalemia was mainly seen with nonselective rather than with cardio-selective beta-blockers [2]. However, there is one reported case of atenolol-induced hyperkalemia in the medical literature [4]. Severe hyperkalemia as a complication of timolol, a topically applied beta-blocker, has also been reported [2]. The duration of hyperkalemia can be fairly prolonged with advanced chronic kidney disease, particularly when renally cleared beta-blockers such as atenolol, are administered [2].

In addition to discontinuing medications that can increase potassium and removal of dietary sources of potassium intake, available treatments for hyperkalemia include intravenous calcium to ameliorate cardiac toxicity if present, intravenous insulin, glucose, nebulized beta-adrenergic agonist, oral gastrointestinal cation exchangers, loop diuretics, and emergency hemodialysis as a last resort for patients with severe renal impairment or for patients with potentially lethal hyperkalemia not responding to conservative measures [8].

This patient is reported to be compliant with hemodialysis. There was no significant modification of his diet over the past years as per the patient. This is his fourth presentation with hyperkalemia, the last two times also assumed to be provoked by the reintroduction of atenolol when studied retrospectively. Every time upon discharge, he was always lost to follow up and presented with a similar problem. Upon looking back into his past records, atenolol was discontinued every time, and he was explained the reason, but it was again prescribed by different doctors a few days before his presentation to the ED. Upon discharge this time, his dedicated primary care physician was contacted and explained the situation, and the patient was also counseled to follow up with a particular primary care physician for comprehensive care.

Because of the absence of any documented underlying cardiac disease and the correlation of recent reintroduction of beta-blocker, we would like to propose that type II second degree heart block was caused by atenolol-induced hyperkalemia. Albeit atenolol can cause heart block as well; AV block is commonly related to drugs but rarely caused by drugs [9].

## Conclusions

Even though ESRD patients are already very susceptible to hyperkalemia, in our patient who is compliant to hemodialysis, we should also look into other possible causes of this electrolyte disturbance. Beta-blockers are commonly used for the treatment of hypertension. Physicians should be aware of the medications that can precipitate hyperkalemia, patient characteristics with a higher risk of hyperkalemia, the range of dysrhythmias attributed to hyperkalemia, and implementing treatments without waiting for lab results if necessary.
